# Association between neighborhood disadvantage and children's oral health outcomes in urban families in the Chicago area

**DOI:** 10.3389/fpubh.2023.1203523

**Published:** 2023-06-29

**Authors:** Helen H. Lee, John J. Dziak, David M. Avenetti, Michael L. Berbaum, Yuwa Edomwande, Margaret Kliebhan, Tong Zhang, Karla Licona-Martinez, Molly A. Martin

**Affiliations:** ^1^Department of Anesthesiology, College of Medicine, University of Illinois Chicago, Chicago, IL, United States; ^2^Institute for Health Research and Policy, University of Illinois Chicago, Chicago, IL, United States; ^3^Department of Pediatric Dentistry, College of Dentistry, University of Illinois Chicago, Chicago, IL, United States; ^4^Department of Pediatrics, College of Medicine, University of Illinois Chicago, Chicago, IL, United States

**Keywords:** neighborhood, oral health, childhood caries, social determinants of health, plaque

## Abstract

**Purpose:**

The prevalence of childhood caries in urban Chicago, compared with national and state data, indicates that neighborhood context influences oral health. Our objective was to delineate the influence of a child's neighborhood on oral health outcomes that are predictive of caries (toothbrushing frequency and plaque levels).

**Methods:**

Our study population represents urban, Medicaid-enrolled families in the metropolitan Chicago area. Data were obtained from a cohort of participants (child–parent dyads) who participated in the Coordinated Oral Health Promotion (CO-OP) trial at 12 months of study participation (*N* = 362). Oral health outcomes included toothbrushing frequency and plaque levels. Participants' neighborhood resource levels were measured by the Area Deprivation Index (ADI). Linear and logistic regression models were used to measure the influence of ADI on plaque scores and toothbrushing frequency, respectively.

**Results:**

Data from 362 child–parent dyads were analyzed. The mean child age was 33.6 months (SD 6.8). The majority of children were reported to brush at least twice daily (*n* = 228, 63%), but the mean plaque score was 1.9 (SD 0.7), classified as “poor.” In covariate-adjusted analyses, ADI was not associated with brushing frequency (0.94, 95% CI 0.84–1.06). ADI was associated with plaque scores (0.05, 95% CI 0.01–0.09, *p* value = 0.007).

**Conclusions:**

Findings support the hypothesis that neighborhood-level factors influence children's plaque levels. Because excessive plaque places a child at high risk for cavities, we recommend the inclusion of neighborhood context in interventions and policies to reduce children's oral health disparities. Existing programs and clinics that serve disadvantaged communities are well-positioned to support caregivers of young children in maintaining recommended oral health behaviors.

## Introduction

Pediatric oral health disparities by race/ethnicity in the United States are an enduring problem that is rooted in social determinants of health and structural racism (1, 2). The social ecological model is a useful framework for operationalizing protective and risk-related factors associated with these disparities (3, 4). While predictive factors of oral health disparities have been measured at public health levels (access to community water fluoridation, access to care) (5), interpersonal levels (insurance status, family income, family environment) (6, 7), and individual levels (child toothbrushing habits and dietary intake) (8–10), less is known about the influence of neighborhood on oral health.

Neighborhood context is increasingly recognized as an important predictor for health disparities, as living in predominantly minoritized communities is associated with an imbalance of protective factors (access to providers for preventive and timely care, sources of nutrient-rich foods, safe recreational areas) vs. detracting factors (environmental violence, exposure to unhealthy foods, tobacco, and alcohol) that influence health (11, 12). This point is salient because poor and minoritized populations often reside in areas deprived of resources such as access to food, safety, education, employment, and quality housing. Populations living in areas of high disadvantage experience elevated rates of systemic diseases, utilize more health services and experience severe health outcomes, such as maternal mortality (13). Where an individual lives may be as important as, and likely linked to, *how* an individual lives. While the prevalence of dental caries in young children has declined over time nationally, oral health disparities remain. The oral health goals of the Healthy People 2020 program appear within reach on a national level, but not for children who reside in Chicago. The prevalence of dental caries among 6–9-year-old children differs when comparing the Healthy People 2020 target (< 49%) to the prevalence at the national level (54.4%), in Illinois (53%), and in Chicago (65%). Similarly, the prevalence of young children with untreated caries is higher for those who reside in Chicago compared with state and national cohorts (14).

Although the role of neighborhood context on children's oral health is not well studied, we have observed poor oral health outcomes in children who reside in urban Chicago neighborhoods (15–17). Coordinated Oral Health Promotion (CO-OP) Chicago was a cluster-randomized controlled trial that tested the impact of community health workers (CHWs) on child toothbrushing behaviors in urban, low-income children under 3 years of age. The CHW-led oral health intervention consisted of up to four household visits over 12 months to provide oral health education, social support, and navigational assistance. The main clinical trial findings did not demonstrate a significant intervention impact on children's oral health outcomes (neither toothbrushing frequency nor plaque levels) (17). This cross-sectional analysis presented here aimed to delineate the influence of a child's neighborhood on the toothbrushing frequency and plaque levels in the CO-OP Chicago cohort.

## Methods

### Study population

Data were derived from the CO-OP Chicago cluster-randomized trial (NCT03397589) that tested the effect of a community health worker-led intervention on children's toothbrushing behaviors. The main findings from the clinical trial revealed no differences in outcomes between participants in the intervention and control arms (17). Therefore, for this analysis, participants were considered a single cohort. Recruitment targeted healthy, low-income children and their families in Cook County, Illinois. Participants (420 child-caregiver dyads) were recruited from Special Supplemental Nutrition Program for Women, Infants, and Children (WIC) centers and pediatric medical clinics from January 2018 through February 2019. Children were 6–36 months of age (mean 21.5, SD 6.9) at the time of enrollment and had at least two teeth. Caregivers were at least 18 years of age, spoke English or Spanish, and served as the primary caregiver of the child. Caregivers identified themselves as Black (41.9%) or Hispanic (52.1%). Other sociodemographic characteristics of CO-OP participants have been previously reported (16, 18). Children with medical conditions that could limit their ability to participate in study activities were excluded. Research assistants (RAs) collected data at baseline, 6, and 12 months after enrollment. Data collection was conducted in either homes or a participant-preferred venue and consisted of caregiver self-report, clinical assessment, and observation of brushing behaviors (12).

### Variables

CO-OP data included in this study are child and caregiver demographics, insurance status, and oral health outcomes.

#### Oral health outcomes

RAs asked caregivers about their child's toothbrushing frequency, which we coded as twice a day or more vs. less than twice a day because twice daily brushing is recommended. Plaque levels were obtained from images of children's primary maxillary incisors that had been painted with a plaque-disclosing solution. Plaque scores were based on images of teeth with a plaque-disclosing solution using the Oral Hygiene Index—Maxillary Incisor Simplified. This is designed to be an ecological momentary assessment of plaque condition. Families are not advised to either brush or not brush to avoid coaching them one way or the other. When scoring images, thin biofilm (develops in a matter of hours) was not scored. Plaque accumulation, indicating a lack of effective brushing, was scored. Research assistants obtained photographic images of child participants in their home environment in the context of collecting other data related to oral health behaviors. Research assistants and research staff were responsible for scoring and underwent extensive calibration (19). Because data collection times were scheduled at the convenience of the study participants, the study protocol could not standardize the timing of plaque-disclosing solution application. This is a conventional approach to plaque disclosing. Details regarding plaque scores are included in the publication of the main clinical trial (17). The images were scored by calibrated clinicians using the Oral Hygiene Index—Maxillary Incisor Simplified (OHI-MIS) (20). Plaque scores were categorized as good (< 0.7), fair (0.7–1.8), and poor (>1.8–3.0). Because all children experienced ongoing tooth eruption and the potential for more plaque increased over time, we included plaque scores collected over 12 months of study participation to capture the most potential scores.

#### Neighborhood risk

Neighborhood indices can be useful tools for research into sociobiologic mechanisms of disease and can also help inform policy and health system-level interventions to better align resources with needs. The Area Deprivation Index (ADI) measures socioeconomic disadvantage among neighborhoods using data from the American Community Survey (21, 22). The 2019 ADI (v3.0) was constructed using the 2015–2019 American Community Survey Five-Year Estimates. The ADI is a composite of the following domains: income, education, employment, and housing quality. The ADI has been recommended to identify target neighborhoods for program delivery. For research purposes, the ADI has been used as an explanatory variable in health disparities research (23). On a health system level, the ADI serves as an addition to other social risk factors to better understand population health and health system performance (24). The ADI is reported as percentiles on the national level. On the state level, ADI scores are split into 10 equal sections and reported as decile groups.

An ADI state decile of 1 represents the least disadvantaged neighborhood, and 10 represents the most disadvantaged one. The ADI utilizes census block groups as the most stable unit of the neighborhood. Census block group shapefiles were downloaded from the US Census Bureau (25). For our analysis, participants' locations were geocoded with residential information (recorded at the study baseline) using Google Maps and the GPS Visualizer converter tool (26). Each participant's address was linked to latitude and longitude coordinates (*N* = 362) and then categorized into the correlating Illinois census block. Illinois census blocks (1–10) were then matched with the 2019 area deprivation index (ADI).

#### Covariates

We collected child and caregiver demographic information related to age (years), reported sex (men, women), race and ethnicity, residential address, and caregiver education levels (more than high school, high school/general educational development test, or less than high school graduate).

#### Visualizing participant neighborhoods

ArcGIS Pro 2.7 was used to layer CO-OP Chicago participant data over ADI data. The 2019 state ADI deciles were plotted for census block groups within Chicago and its neighboring western and southern suburban areas. Overlapping participant data points were plotted using the ArcGIS tool Disperse Markers with the following parameters: a point size of 4, a reference scale of 1:250,000, an expanded dispersal pattern, and a minimum spacing of 0 points. Census shapefiles and participant data points were plotted alongside Chicago city boundaries and on top of ArcGIS's World Topographic Map and World Hillshade for geographical reference (27–29).

#### Human subjects

Institutional Review Boards at the University of Illinois at Chicago [2017-1090], the University of California San Francisco [16-19920], and the Chicago Department of Public Health [16–06] approved the trial. Caregivers provided written informed consent. Trial oversight was also provided by an NIH Data Safety Monitoring Board, an external monitor reporting to the funder, and a UIC Community Advisory Board.

### Analysis

Toothbrushing frequency was treated as a dichotomous variable (< twice per day vs. at least twice per day). The plaque score was treated as a continuous variable. We used linear regression models to assess the association between ADI and plaque score. Logistic regression models were used to assess the association between ADI and children's brushing frequency. Because the ADI variable represented sociodemographic characteristics at the neighborhood level, we also included individual-level demographic covariates: child age, child sex, caregiver education (high school degree/equivalent or less than high school degree), and caregiver race/ethnicity (non-Hispanic black vs. non-Hispanic Other, Hispanic vs. non-Hispanic Other).

## Results

**Figure 1:**
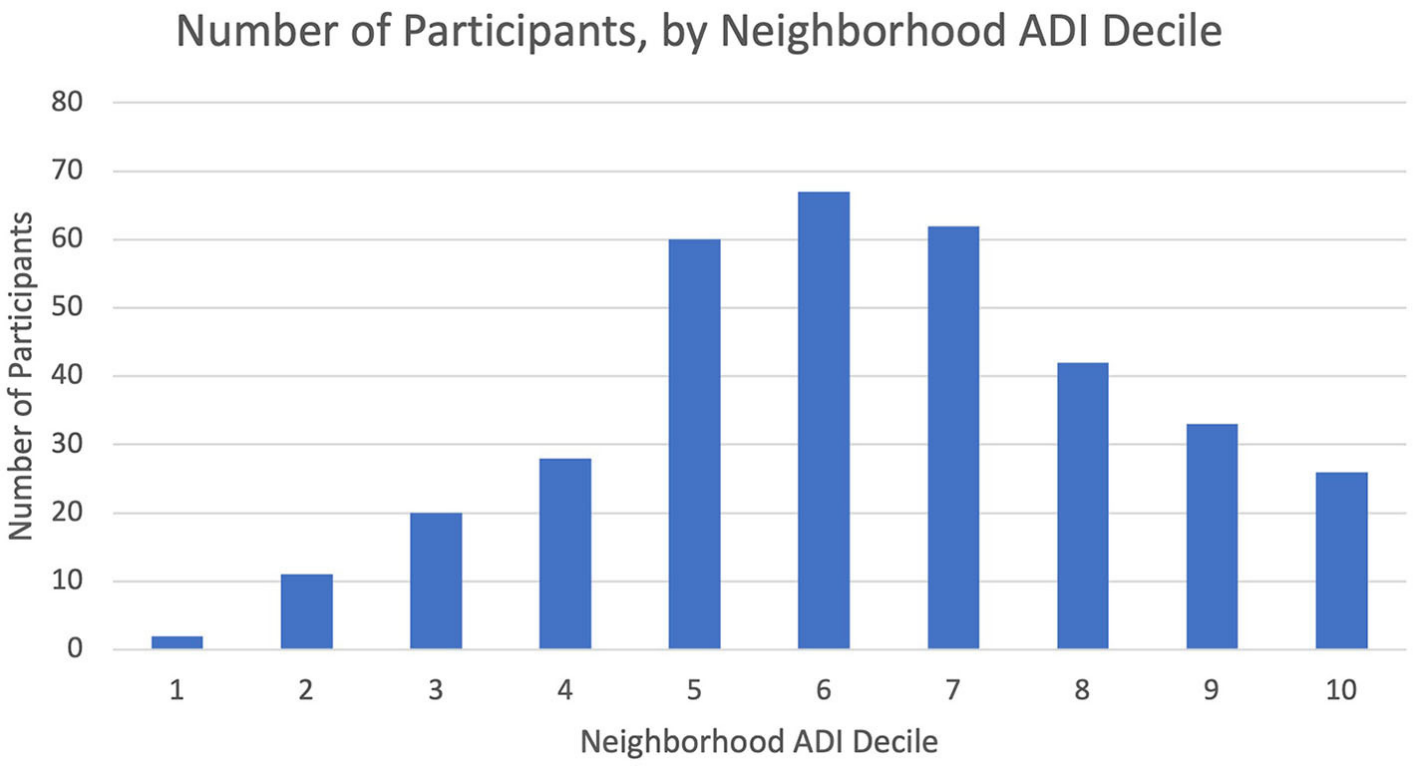
Number of participants by state ADI decile of their residential neighborhood. Organization of CO-OP participants' residential neighborhoods with state-level Area Deprivation Index (ADI) deciles. Higher ADI deciles represent greater degrees of neighborhood resource deprivation.

**Figure 2:**
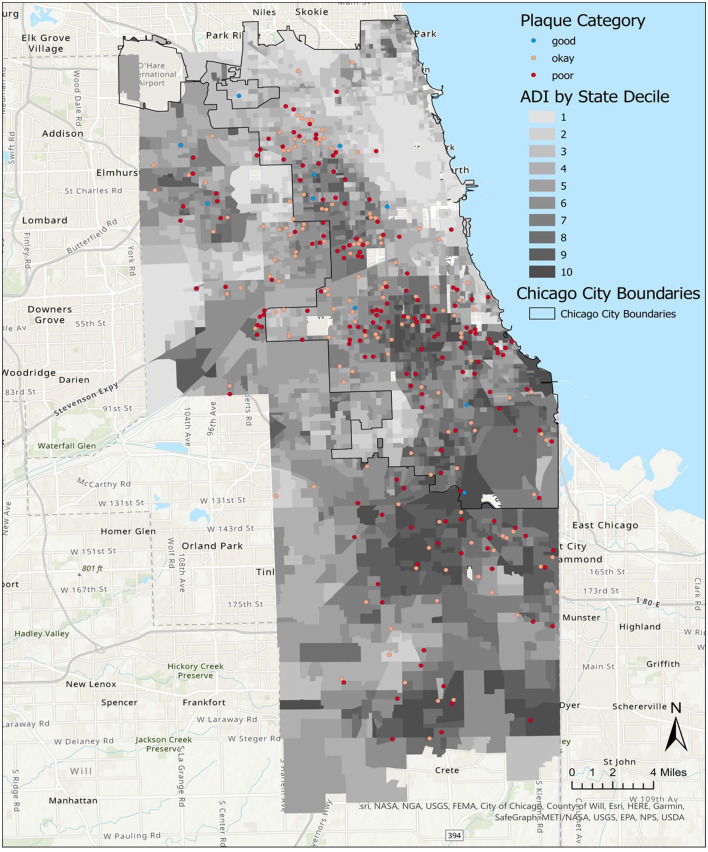
Distribution of child brushing frequency in Chicago neighborhoods, by Area Deprivation Index (ADI). A map of Chicago neighborhoods characterized by the ADI. State ADI deciles indicate increasing severity of neighborhood resource deprivation as scores increase from 1 to 10.

We analyzed data representing 362 participants who had completed CO-OP data collection at 12 months of study participation (Table 1). Most participants lived in neighborhoods categorized as ADI>6 (Table 1, Figure 1). We did not observe any differences in demographics or ADI scores between participants at baseline vs. those who completed data collection at 12 months. We did not observe a visual pattern in the distribution of children's neighborhoods and brushing frequency (< 2 times a day vs. at least 2 times a day) (Figure 2). Neighborhood deprivation was not significantly associated with a child's brushing frequency (estimated odds ratio 0.94, 95% CI 0.84–1.06, *p* value = 0.30) (Table 2).

**Table 1:** Characteristics of child CO-OP participants^*^.

**Child**	
Age at baseline, mean months (SD)	21.6 (6.8)
Current age at 12 months in Study (SD)	33.6 (6.8)
Sex	*n* (%)
Female	192 (53.0)
Male	170 (47.0)
Race/Ethnicity	*n* (%)
Non-Hispanic other	24 (6.6)
Non-Hispanic black	151 (41.7)
Hispanic	187 (51.7)
Brushing frequency	*n* (%)
Twice a day or more	228 (63.0)
Less than twice a day	134 (37.0)
Plaque score, mean (SD)	1.9 (0.7)
	*n* (%)
Good (< 0.7)	53 (14.6)
Fair (0.7–1.8)	143 (39.5)
Poor (>1.8)	166 (45.8)
**Caregiver**	
Education	*n* (%)
More than high school	198 (54.7)
High school/GED	114 (31.5)
Less than high school	50 (13.8)
**Neighborhood (Area of Deprivation Index, state decile rank)**, ***n*** **(%)**	
**Decile rank (higher** = **more deprived)**	
1	2 (0.6)
2	11 (3.1)
3	20 (5.7)
4	28 (8.0)
5	60 (17.1)
6	67 (19.1)
7	62 (17.7)
8	42 (12.0)
9	33 (9.4)
10	26 (7.4)

^*^Data reflects CO-OP participants who completed study data collection activities at month 12. Total sample size *N* = 362. Mean ADI decile rank = 6.3, SD 2.1. Plaque scores range 0–3. Plaque scores were categorized as good (< 0.7), fair (0.7–1.8), and poor (1.9–3.0).

**Table 2:** Logistic regression between neighborhood deprivation and brushing at least twice a day.

**Variable**	**b**	**SE(b)**	**Odds ratio**	**95% CI for odds ratio**	***p* value**
**Intercept**	**−0.46**	**0.68**	**0.63**		
**ADI, state decile**	−0.06	0.06	0.94	0.84–1.06	0.30
Child Age, months	0.04	0.02	1.04	1.01–1.07	0.02
Child sex, female (1) vs. male (0)	0.15	0.11	1.34	0.87–2.09	0.19
Caregiver education, HS/GED (1) vs. more than HS (0)	−0.10	0.17	0.86	0.53–1.41	0.57
Caregiver education, < HS (1) vs. more than HS (0)	0.05	0.22	0.99	0.51–1.96	0.83
Caregiver race, non-hispanic black (1) vs. non-Hispanic other (0)	0.13	0.20	1.38	0.56–3.44	0.50
Caregiver ethnicity, Hispanic (1) vs. non-Hispanic other (0)	0.09	0.19	1.44	0.57–3.62	0.62

*n* = 351. A multiple logistic regression model was used to test the association between a child's neighborhood context and likelihood of brushing at least twice a day. ADI, Area Deprivation Index. On the state level, ADI scores are split into 10 equal sections and reported as decile groups. Lower state decile represent the least disadvantaged neighborhood. The highest level of caregiver education was categorized in terms of whether participants had to completed high school (HS) or graduate equivalent degree (GED).

**Figure 3:**
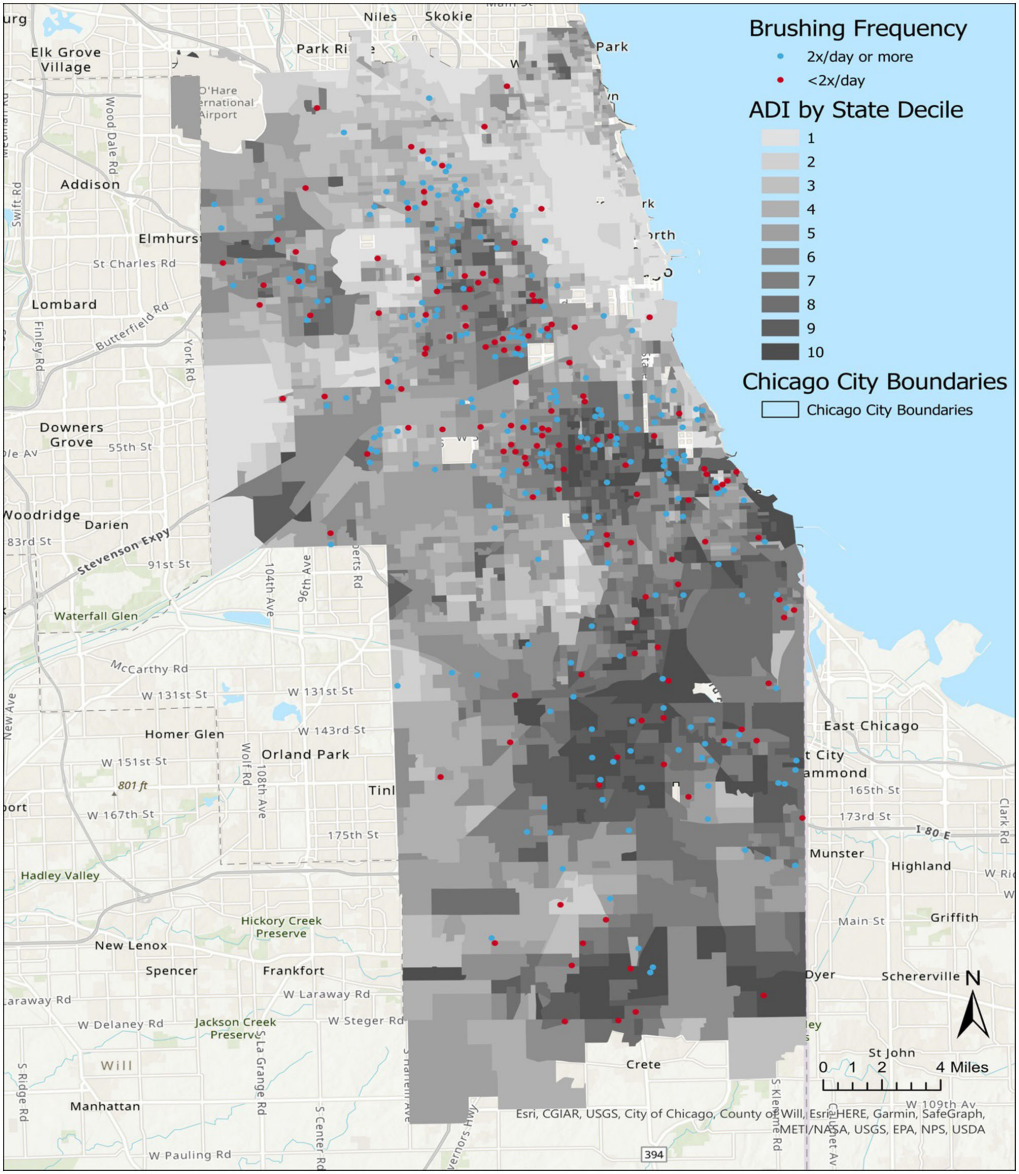
Distribution of child plaque levels in Chicago neighborhoods, by Area Deprivation Index (ADI). A map of Chicago neighborhoods characterized by the ADI. State ADI deciles indicate increasing severity of neighborhood resource deprivation as scores increase from 1 to 10.

The average plaque score of child participants was 1.9 (SD 0.7), with scores between 1.9 and 3.0. Plaque scores between >1.8 and 3.0 were categorized as poor. The visual distribution of children with “good” plaque scores (< 0.7) appeared to be primarily in neighborhoods with ADI deciles 1–5 (Figure 3). Covariate-adjusted analysis revealed that a higher ADI decile was associated with a higher average child's plaque score (regression coefficient 0.05, 95% CI 0.01–0.09, *p* value = 0.007) (Table 3).

**Table 3:** Regression of child plaque levels on neighborhood deprivation.

**Variable**	**b**	**SE (b)**	**95% CI**	***P* value**
**Intercept**	**1.50**	**0.25**		
**ADI, state decile**	0.05	0.02	0.01–0.09	0.007
Child age (months)	−0.003	0.005	−0.014–0.007	0.55
Child sex, female (1) vs. male (0)	0.03	0.07	−0.11–0.18	0.64
Caregiver education, HS/GED (1) vs. >HS (0)	0.08	0.08	−0.08–0.24	0.34
Caregiver education, < HS (1) vs. >HS (0)	0.21	0.11	−0.005–0.43	0.06
Caregiver race, non-Hispanic black (1) vs. non-Hispanic other (0)	0.04	0.15	−0.26–0.33	0.80
Caregiver ethnicity, Hispanic (1) vs. non-Hispanic (0)	0.13	0.15	−0.16–0.42	0.37

*N* = 310, R^2^ = 0.05. A multiple linear regression model was used to test the association between a child's neighborhood context and plaque levels. ADI, Area Deprivation Index. On the state level, ADI scores are split into 10 equal sections and reported as decile groups. Lower state decile represent the least disadvantaged neighborhood. The highest level of caregiver education was categorized in terms of whether participants had completed high school (HS) or graduate equivalent degree (GED).

## Discussion

Children's oral health reflects factors that operate at the individual, household, neighborhood, and policy levels. We have studied a population of largely urban, Medicaid-enrolled families in the Chicago metropolitan area. Within this population at high risk for poor oral health, we were interested in the influence of neighborhood on children's oral health outcomes. The Area Deprivation Index (ADI) allows for the analysis of multiple social determinants of health that operate at the neighborhood level in both positive and negative directions. It is important to understand how neighborhood-level vs. individual-level behaviors and poverty influence a child's oral health because these distinctions can inform directed policy and resource allocation. It is essential to understand how neighborhood context, as a composite of multiple social determinants of health, might influence a child's oral health status. The ADI did not statistically significantly predict a child's brushing frequency in this sample. However, we did observe an association between high degrees of neighborhood deprivation and worse plaque levels in children.

In young children, brushing frequency and quality are tied to the physical presence of an adult. Public Health England provides guidance on parentally-supervised brushing (PSB) for children up to 8 years of age (30–32). This is based on evidence that parental supervision ensures the effective removal of plaque as a measure of the quality of a child's brushing behaviors (32). PSB incorporates not only brushing frequency, duration, and use of fluoridated toothpaste but also acknowledges the complex dynamics between a caregiver and child during brushing. The presence of regular, parent-assisted brushing is predictive of better child oral health outcomes, including caries (33). Caregiver assistance in a child's brushing activity can be hindered by activities of daily living and stressors on caregiver time (16, 17). We anticipated that the caregiver's daily life stressors would be influenced by neighborhood context, which would then translate into a child's brushing frequency. However, we did not find a statistically significant relationship between brushing frequency and neighborhood context. Our findings may reflect the greater influence of parent–child dynamics and factors that operate at the household level.

Our findings associated with high ADI levels and worse child plaque scores coincide with the pattern of caries in urban Chicago neighborhoods. Plaque levels reflect the dynamics between the intake of fermentable carbohydrates and the quality of toothbrushing behaviors, which can reduce plaque buildup. In this sense, plaque scores could serve as a proxy for dietary intake and quality toothbrushing in young children. While the ADI was not associated with brushing frequency, future analyses could address other factors related to brushing quality, characterized by duration (2 min), caregiver assistance, use of fluoride toothpaste, and brushing and tooth–gum interface. The clinical significance of high plaque levels is that high plaque levels lead directly to the formation of tooth decay. Plaque levels are influenced by formation factors (eating and drinking habits) and removal factors (effective brushing habits, saliva). Obtaining plaque scores may be logistically easier than determining the presence of caries for studies that are based in the community, as plaque images can be obtained by trained research staff and the interpretation of the images, by trained clinicians, can be centralized (17). Additionally, our findings support the use of the ADI as a useful summary measure of multiple social determinants of oral health at the neighborhood level.

Positive and negative findings should be interpreted through the lens of our study limitations. First, we acknowledge that our study sample is not a representative sample of Chicago neighborhoods. This study is a secondary analysis of data from a trial focused on a primarily Medicaid-enrolled population. Residential areas of study participants are skewed toward neighborhood with higher ADI levels, which likely reflects the sources of recruitment. However, we still observed participants residing in a full range of neighborhood deprivation (ADI state deciles ranged from 1–10). It is possible that data from a population that represents greater socioeconomic diversity would yield different results in terms of the relationship between ADI and child brushing frequency. Second, the ADI measure is derived from census data, which may not adequately account for undocumented immigrants. In a previous study, we reported caregiver concerns that relate to undocumented status (34), thus suggesting possible challenges in understanding the role of neighborhood context with the ADI for our study sample.

In summary, our findings that neighborhood context influences a child's oral health status are similar to prior health disparities studies (35, 36). While brushing frequency was not associated with neighborhood ADI in the context of our study, our findings support that neighborhood-level factors are associated with plaque levels in children that place them at high risk for developing cavities. Connecting populations at high risk for the disease to timely interventions is a cornerstone of public health efforts. While modest, our findings should encourage researchers to continue further studies using the ADI to identify children at high risk for severe cavities in other geographic areas of the United States. Understanding the relationship between the ADI and children's oral health can inform ongoing efforts by stakeholders at the community level (e.g., faith-based organizations) and health systems (e.g., emergency departments that have high rates of toothache visits). Medical and dental care is constrained by structural inequities. Decisions about the equitable allocation of resources and access to care are challenging, but neighborhood-based algorithms appear to acknowledge some of the sources of disparities in healthcare. Health systems and institutions such as the University of Pittsburgh Medical Center and the Commonwealth of Pennsylvania have incorporated the ADI as a tool in rationing scarce COVID-19 medications (37). On a population level, policy- and community-based efforts should address access to healthy foods, clean fluoridated drinking water, and interventions to support families in maintaining healthy eating and drinking habits. Because the neighborhood context is important, the role of community-based interventions should be prioritized. Directed resources should address knowledge issues, such as demonstrating the quality of adult assistance with brushing activities and enhancing awareness and child-specific strategies to increase the consumption of healthy foods and beverages. Leveraging upon existing programs that serve high-risk communities in high-ADI neighborhoods, such as WIC clinics or federally qualified health centers, particularly those that provide medical and dental care, may yield the greatest impact.

## Data availability statement

The data analyzed in this study is subject to the following licenses/restrictions: Data and other products of the study will be made publicly available, concordance with governance policies and protocols. Requests to access these datasets should be directed to mollyma@uic.edu.

## Ethics statement

The studies involving human participants were reviewed and approved by Institutional Review Boards at the University of Illinois at Chicago [2017-1090], University of California San Francisco [16-19920], and Chicago Department of Public Health [16–06] approved the trial. Written informed consent to participate in this study was provided by the participants' legal guardian/next of kin.

## Author contributions

HL, MM, JD, and MB contributed to conception, design of the work, and critical revisions of the manuscript. HL was the lead writer in all drafts of the manuscript. DA, YE, and MK contributed to data collection. MB, JD, and TZ performed statistical analyses. HL, MK, KL-M, and MM wrote sections of the manuscript. All authors contributed to manuscript revision, read, and approved the submitted version.
